# Floral mechanisms promote pollination success and reduce the incidence of self‐pollination in a fly‐pollinated self‐incompatible orchid

**DOI:** 10.1002/ece3.11295

**Published:** 2024-04-24

**Authors:** Sheng Zhang, Shi‐Mao Wu, Jiang‐Yun Gao

**Affiliations:** ^1^ Institute of Biodiversity, School of Ecology and Environmental Science, Yunnan University Kunming Yunnan China

**Keywords:** breeding system, *Bulbophyllum funingense*, fly pollination syndrome, Orchidaceae

## Abstract

Among flowering plants, self‐incompatibility is considered the most efficient system for avoiding self‐fertilization. However, many self‐incompatible plants have also evolved floral mechanisms to reduce sexual conflict. In China, some studies of *Bulbophyllum* have been reported to be self‐incompatible and no fruit sets. However, we have observed relatively high fruit sets in *Bulbophyllum funingense*. Therefore, we speculated that if *B. funingense* is also self‐incompatible, and it might present a floral mechanism to avoid sexual conflict. Natural fruit sets, pollinia removal and deposition rates were determined and breeding system was tested in a hand‐pollination experiment. The pollination process and visiting frequency of pollinators and their behavior after escape from access were observed and recorded. Floral traits associated with pollination and pollinator size were measured. *B. funingense* was completely self‐incompatible, the fruit sets of cross‐pollination in 2 years were all more than 70%, and the natural fruit sets for 2 years were 1.70 ± 4.31% and 6.63 ± 5.29%, respectively. *B. funingense* did not produce strong odor or nectar, but produced a kind of secretions from its labellum that attracted flies. *Calliphora vicina* (Calliphoridae) was its only effective pollinator. When *C. vicina* licked the secretions, they were stuck in the access for a long time. Thus, when they escaped from access, they almost always flew quickly away from the inflorescence removing pollinia most of the times. In *B. funingense*, a floral mechanism improves pollinia transfer efficiency, reduces pollinia waste, promotes pollination success, reduces the incidence of self‐pollination, and avoids sexual conflict to a certain extent.

## Graphical abstract

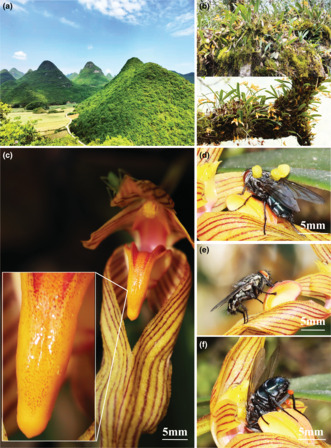

## 1 INTRODUCTION

Darwin interpreted the various pollination mechanisms of plants as the means of increasing outcrossing (Darwin, 1859). Plants have evolved numerous mechanisms to avoid self‐pollinating, thereby reducing inbreeding depression (Narbona et al., 2011). Self‐incompatibility is considered the most efficient system for avoiding self‐fertilization (Takayama & Isogai, 2005), but many self‐incompatible plants have also evolved floral mechanisms to reduce sexual conflict anyways; for example, the separation of male and female functions is thought to reduce self‐fertilization and promote outcrossing (Lloyd & Webb, 1986; Webb & Lloyd, 1986).

The majority of orchids present pollination systems, such as deceptive pollination, that promote outcrossing (Jersáková et al., 2006; Johnson et al., 2003), and they have a wide variety of floral designs and displays that maximize this (Jin et al., 2012). Most orchids exhibit pollination specificity, with approximately 67% of orchids having only one recorded pollinator (Johnson & Steiner, 2000; Scopece et al., 2010; Tremblay, 1992). Therefore, these species with pollination specificity are often pollinator‐limited (Nilsson, 1992; Scopece et al., 2010; Tremblay, 1992). Of course, other factors can also lead to pollinator‐limited, such as pollinia quality and pollinator visit frequency (Tremblay et al., 2005). The natural fruit set is generally low in non‐autogamous orchids (Tremblay et al., 2005). Among them, the fruit set is often extremely low in self‐incompatible orchids (Tremblay et al., 2005), such as the fruit set of *Tolumnia variegata*, which is less than 2% (Ackerman et al., 1997; Calvo, 1993), and the 3‐year average fruit set of *Coelogyne fimbriata*, which is only 3.62% (Cheng et al., 2009).

Orchids have a variety of pollinators, including birds, moths, butterflies, a wide variety of flies, numerous bees and wasps (Ackerman et al., 2023; van der Pijl & Dodson, 1966). Myophily (pollination by flies) is the second most common pollination syndrome in Orchidaceae (Christensen, 1994; Peter, 2011; van der Pijl & Dodson, 1966). Fly‐pollinated orchids usually utilize a range of flower traits, such as dull‐colored flowers and foul odor, to attract flies (Humeau et al., 2011; van der Pijl & Dodson, 1966), and a few species emit fragrant or fruity scent to attract flies (Tan et al., 2002; Tan & Nishida, 2000). Of course, some fly‐pollinated orchids can also produce nectar (e.g., *Epidendrum tridactylum*, Pansarin & Pansarin, 2014).

*Bulbophyllum* species are thought to exhibit the classic fly pollination syndrome (Dressler, 1981, 1993; POWO, 2021). The “hinged labellum” of *Bulbophyllum* orchids is considered important, and its role in pollination was first described in *B. macranthum*, in which the weight of the insect causes the labellum to move downward, but when the insect crawls along the labellum to reach an balance point, the labellum bounces back and hurls the pollinator toward the column (Ridley, 1890). Previous studies of *Bulbophyllum* species, such as *B. weddellii*, *B. invoIutum* and *B. ipanemense*, described them as self‐compatible (Borba et al., 1999). However, *B. ambrosia* has been reported to be self‐incompatible (Chen & Gao, 2011). In a study in China, *B. ambrosia* produced a fragrance while providing nectar to attract *Apis cerana cerana* to pollinate, but self‐incompatibility results in a low rate or lack of natural fruits (Chen & Gao, 2011). In another study, an obligately outcrossing epiphytic orchid, *B. bicolor*, emitted strong fecal odors to attract *Neomyia claripennis* (Muscidae) for pollination. Surprisingly, a preponderance of clonality triggers loss of sex in *B. bicolor*, resulting in its lack of fruiting for years (Hu et al., 2017).

Like the above two species, *B. funingense* has abundant clonal growth in limestone habitats, yet natural fruits have been observed. Thus, we set out to answer the following three questions: (1) What species is the pollinator of *B. funingense*? What attracts pollinators? (2) Is *B. funingense* also self‐incompatible? (3) Is there any floral mechanism to reduce sexual conflict?

## 2 MATERIALS AND METHODS

### 2.1 Study site and species

Our study site was at a karst mountain top in WanFengShan Nature Reserve (WNR) (24°51′28″ N, 104°29′12″ E; altitude 1585 m), which is located in Luoping County, Yunnan Province. We found 78 orchid species from 35 genera (Liu et al., 2023), and in our previous investigation, we had found some *Bulbophyllum* species in WNR.

**FIGURE 1:**
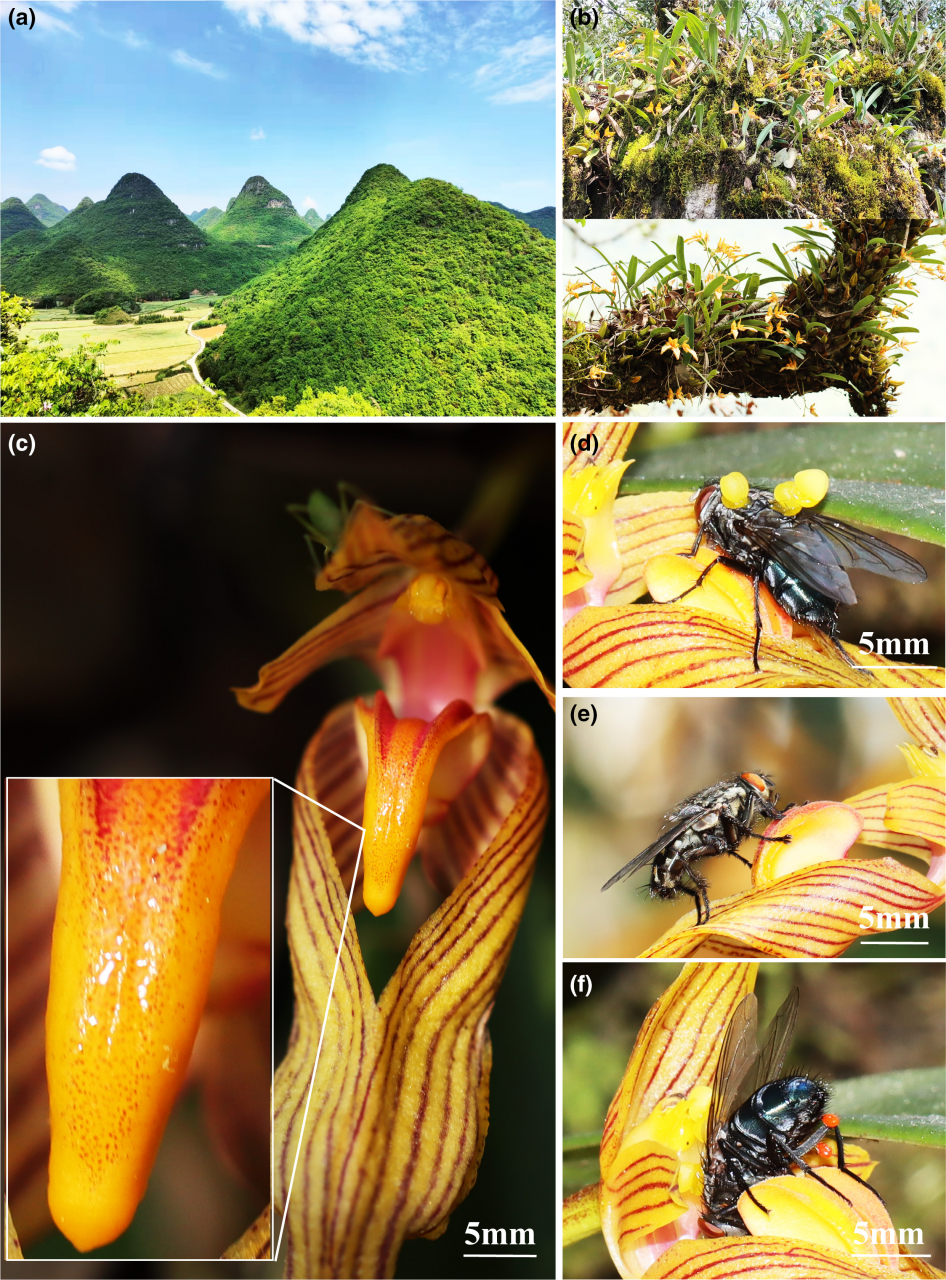
Karst independent mountains, two habitats, flower (secretion of lip), the different visitors of *Bulbophyllum funingense*. (a) Study site: independent mountains in WNR; (b) Two habitats: stone habitat and tree habitat; (c) Flower (secretion of lip); (d) *C. vicina* licking secretion; (e) *S. carnaria* licking secretion; and (f) *C. vicina* stuck in access.

*Bulbophyllum funingense* is an epiphytic orchid in the *Bulbophyllum* genus and is endemic to China. It was first found in Funing County in southeastern Yunnan Province and grows in limestone habitats and tree habitats in karst landforms. In the WNR, *B. funingense* is widely found attached to rocks or tree trunks on limestone mountains and is the dominant orchid species (Figure 1a,b).

### 2.2 Flowering phenology and morphology

During flowering periods in 2022 and 2023, we randomly labeled 200 inflorescences to count the number of flowers in each inflorescence. Randomly selected 60 flowers in 26 inflorescences were used to measure nectar volume and sugar concentration using a 10‐μL microsampler (Shanghai Anting Microsampler Field, China) and a hand‐held temperature‐compensated refractometer (Eclipse, Bellingham and Stanley Ltd, UK), respectively (Zhou et al., 2016). Because the *Bulbophyllum* orchids usually emit a strong odor, e.g. *B. ambrosia* and *B. bicolor* (Chen & Gao, 2011; Hu et al., 2017), we smell flowers to get an initial sense of whether the flower produced an odor. We also monitored the floral longevity after bagged, self‐pollinated and cross‐pollinated, with 20 inflorescences randomly selected for each treatment.

### 2.3 Hand‐pollinations, natural fruit set, pollinia removal and deposition

To determine the breeding system, we randomly selected flowers with no pollinia removal and deposition to carry out three treatments: (1) bagging, (2) self‐pollination, and (3) cross‐pollination. Then, flowers were bagged with nylon mesh bags to prevent insect revisiting. Pollinia for self‐pollination were selected from the same flower, and pollinia for cross‐pollination were taken from another population 50 m away. The experiment was carried out in 2022 and repeated in 2023, 27 flowers were selected for each treatment in 2022 and 50 flowers were selected for each treatment in 2023. After the end of the flowering periods, the fruit sets of the hand‐pollination treatments were calculated. A total of 1730 flowers in 858 inflorescences in 2022 and 2048 flowers in 1011 inflorescences in 2023 were used to calculate the natural fruit sets, respectively. And 984 flowers in 500 inflorescences in 2023 were randomly selected to calculate pollinia removal and deposition ratios.

### 2.4 Floral visitor observations

Observations of pollinating insects were conducted from April 16 to 25, 2022, and March 31 to April 9, 2023. About 200 fully opened flowers of 108 inflorescences were randomly selected and observed on sunny or cloudy (but not raining) days from 6:00 to 18:00. Pollination behavior was observed at a distance of 2 m to avoid human odor and movement disturbance of visiting insects (Pakum et al., 2019). The pollinator's visiting behavior, visiting frequency, single flower residence time and behavior after visiting flowers were recorded. Photos were taken through a digital camera (Canon 90D) with a macro lens (focal length 100 mm) to record the visiting process. After finished all observations, insect nets were used to catch insects during their visiting to flowers, and specimens were processed for species identification. To test the presence of nocturnal pollinators, we randomly labeled 45 flowers of 20 inflorescences, bagged them during the day (6:00–18:00) and unbagged them during night (18:00–6:00), and checked pollinia removal and deposition in the early morning of the next day.

To assess morphological correspondence between floral traits and pollinating insects, we measured traits in 6 flowers, and the size of two kinds of visiting insects, including 6 pollinators and 7 other flies. we measured the distance between the column and labellum (access height), the width of the labellum (access width), the distance from the column foot to the operculum (access length), and the thorax height, thorax width and body length of the pollinating insects (Chen & Gao, 2011; Pakum et al., 2019). Each indicator was repeated three times to reduce the error, and the measurements and sample sizes of each indicator are shown in Table 2.

### 2.5 Data analysis

The Shapiro–Wilk (S‐W) test was used to test the normality of each data group. The Kruskal–Wallis *H* test for *K* independent samples was used to analyze the significant difference in floral longevity, hand‐pollination fruit sets, pollinator body size and floral traits between treatments, and Bonferroni test was used for post hoc tests of all data. At the same time, the Mann–Whitney *U* test was used to analyze the significant difference in cross‐pollination fruit sets, natural fruit sets in different years, and pollinia removal and deposition. The Fisher's exact test was used to examine the pollinator's behavior after escaping from access. All statistical analyses were performed in SPSS ver. 25.0 for Windows. Figures were drawn using Adobe Illustrator ver. 2021 and Adobe Photoshop CS6. The results are expressed as the mean ± standard error (mean ± SE), and the type alpha‐I error is fixed at 5% (thus, all nonsignificant differences have *p* > .05).

## 3 RESULTS

### 3.1 Flowering phenology and morphology

The flowering periods of *B. funingense* took place from late March to mid‐to‐late April. Inflorescence drawn from the base of the pseudobulbs, one inflorescence had 1–4 flowers, with a mean of 2.03 ± 0.56 (*N* = 200). The sepals and petals are orange with several purplish red stripes. The labellum are also orange and has a smooth surface with two longitudinal ridges and purplish‐red spots. The columns are purplish‐red. Like other orchids in the genus, the labellum and column foot form a hinged structure (Figure 1c). Flowers normally lasted for 10.80 ± 1.91 days (ranged from 7 to 15 days) without pollination, and floral longevity was significantly shorted by both self‐ and cross‐pollination (*H* = 43.90, df = 2, *p* < .001), but there was no significant difference in floral longevity between self‐pollination (3.35 ± 0.49 days) and cross‐pollination treatment (3.30 ± 0.47 days; *p* = 1.00 > .05; *N* = 20 inflorescences for each of treatment).

These flowers do not produce any fragrant or foul odor that could be recognized by the human sense of smell, and produce no nectar. However, in sunny weather conditions, a secretion was produced on the labellum. During the noon period every day, a large amount of secretion was produced (Figure 1c). Then, pollinators and other insects were attracted to lick the secretion.

### 3.2 Breeding system, natural fruit set, pollinia removal and deposition rate

The 2‐year fruit set rates for the cross‐pollination treatment were significantly higher than those for the bagging and self‐pollination treatments (*H* = 93.51, df = 2, *p* < .001, Table 1). No fruit was found in the bagging and self‐pollination treatments (*p* = 1.00 > .05, Table 1). The 2‐year cross‐pollination fruit sets were stable with no significant difference between the years (*Z* = −0.46, df = 1, *p* = .64 > .05); in 2022 and 2023, they were 75.00 ± 37.98% and 71.15 ± 35.47%, respectively (Table 1).

**TABLE 1:** Statistics of hand‐pollination and natural fruit set (Mean ± SE).

Year	Hand‐pollination fruit set (%)			Nature fruit set (%)	
	Bagging	Self	Crossing		
2022	0^b^	0^b^	75.00 ± 37.98^a^	2022	1.70 ± 4.31^b^
Inflorescence/flower	13/27	14/27	14/27	Inflorescence/flower	858/1730
2023	0^b^	0^b^	71.15 ± 35.47^a^	2023	6.63 ± 5.29^a^
Inflorescence/flower	24/50	27/50	26/50	Inflorescence/flower	1011/2048

*Note*: The fruit sets of different hand‐pollination treatments were compared with each other, and the natural fruit sets of different years were compared, with different letters indicating significant differences.

The natural fruit set in 2023 was significantly higher than that in 2022 (*Z* = −6.95, df = 1, *p* < .001, Table 1), and these were 1.70 ± 4.31% and 6.63 ± 5.29% in 2022 and 2023, respectively. The pollinia removal was significantly higher than the deposition in 2023 (*Z* = −7.52, df = 1, *p* < .001), which were 32.02 ± 13.65% and 4.04 ± 4.53%, respectively.

### 3.3 Floral visitor observations

During 40 h of monitoring in 2022, no visitors were observed, during the 80 h of monitoring in 2023, both *C. vicina* (Calliphoridae) and *Sarcophaga carnaria* (Sarcophagidae) were observed visiting flowers and licking secretions on the labellum (Figure 1d,e). All flowers that were bagged during the day (6:00–18:00) but unbagged during night (18:00–6:00) remained intact, indicating that no nocturnal insects visited flowers of *B. funingense* outside of our observation time*. C. vicina*'s visits were irregular, but the period of most frequent visiting (approximately 12:00–16:00) almost matched the period of mass secretion production. When *C. vicina* licked the secretions and gradually deepened into access, the hinge labellum derived *C. vicina* into the access, and the access stuck *C. vicina* for a long time (Figure 1f, Video S1). When *C. vicina* escaped from the access, pollinia stuck to *C. vicina*'s thorax and were consequently carried away.

In 2023, we recorded 79 times in which *C. vicina* were stuck in the access, 42 of which were stuck for more than 2 h, and one which died in the access. Thirty‐two times in which they were stuck for between 5 min and 2 h, and only 5 times in which they were stuck for less than 5 min. Of the 79 times *C. vicina* was recorded, 74 times (94%) the fly flew away from the inflorescence immediately when escaping from access, and they all were stuck for a long time. Only 5 of the times, *C. vicina* did not go away from the inflorescence when escaping from access, and all of these were stuck for less than 5 min (*χ* ^2^ = 31.39, df = 2, *p* < .001). In addition, *C. vicina* was recorded 64 times (81%) carrying pollinia when escaping from the access, while the remaining 15 times the fly was not carrying pollinia (*χ* ^2^ = 18.64, df = 2, *p* < .001). In addition, we recorded 26 times that *S. carnaria* were licking the secretions, but they were not stuck or carried pollinia any of these times. Its visiting behavior were almost identical to those of *C. vicina*. However, *S. carnaria* reacted more quickly. When the labellum moved, *S. carnaria* individuals immediately flew away.

The access height significantly differed from thorax height for *C. vicina* and *S. carnaria* (*H* = 19.31, df = 2, *p* < .001, Table 2). The thorax height of *S. carnaria* was significantly higher than the access height (*p* < .001), but there was no significant difference between the access height and thorax height of *C. vicina* (*p* = 1.00 > .05). The access width was significantly wider than the thorax width of *C. vicina* and *S. carnaria* (*H* = 38.75, df = 2, *p* < .001), but there was no significant difference between *C. vicina* and *S. carnaria* (*p* = .36 > .05). Access length was significantly shorter than body length of *C. vicina* and *S. carnaria* (*H* = 49.70, df = 2, *p* < .05).

**TABLE 2:** Measurement of the body structure of visiting insects and floral traits (Mean ± SE).

Traits/species	Samples	Access/thorax height (mm)	Access/thorax width (mm)	Access/body length (mm)
Floral traits	*N* = 6	4.32 ± 0.04^b^	8.18 ± 0.14^a^	8.70 ± 0.14^b^
*Calliphora vicina*	*N* = 6	4.32 ± 0.12^b^	4.67 ± 0.29^b^	10.00 ± 0.16^a^
*Sarcophaga carnaria*	*N* = 7	4.60 ± 0.26^a^	4.43 ± 0.33^b^	11.95 ± 0.56^a^

*Note*: Comparisons are made between floral traits, *C. vicina* and *S. carnaria* indicators (e.g.: Access/Thorax height, Access/Thorax width, Access/Body length), with different letters indicating significant differences.

## 4 DISCUSSION

### 4.1 Flowering phenology and attraction to flies

The floral longevity of *B. funingense* about 10 days, its floral longevity dramatically reduced after pollinated, with its petals withering after about 3 days. Many *Bulbophyllum* species attract fly pollinators by emitting foul odor (van der Pijl & Dodson, 1969), others emitting fragrant odor (Tan et al., 2002; Tan & Nishida, 2000), and these odor are very strong. Unlike those *Bulbophyllum* species, *B. funingense* did not produce nectar or emit any fragrant or foul odor that could be recognized by the human sense of smell. However, it is possible that the odor of the *B. funingense* is not easy to detect, but this led to us to overlook the role of imperceptible volatile odors in attracting pollinators and pollination. More studies are also needed to detect floral odor.

We observed that flies were attracted by secretions from the labellum. The secretion was usually produced in sunny weather and at noon because *B. funingense* grows on the mountaintop. Similarly, *Megaselia* flies have been observed visiting *B. nipondhii* from 15:00 to 17:00 in sunny conditions, which was speculated to be due to *B. nipondhii* exposure to strong sunlight during this period (Pakum et al., 2019). Unfortunately, the secretions are scarce, and quickly evaporate or were reabsorbed. We tried to collect and determine secretions in the field, but it was unsuccessful because the amount produced is very small and difficult to preserve. On the other hand, we collected plants and brought them back to the laboratory, but once plants were collected, they had less secretions than before. There is no doubt that the secretion plays an important role in the pollination process, and we will also study its role in the following studies.

### 4.2 Breeding system, natural fruit set, pollinia removal and deposition

In previous studies in China, both species *B. ambrosia* and *B. bicolor* were reported to be self‐incompatible (Chen & Gao, 2011; Hu et al., 2017), and expected, *B. funingense* is also self‐incompatible. Plants under the bagging and self‐pollination treatments did not produce fruits, indicating that *B. funingense* is dependent on pollination by pollinators and is completely self‐incompatible. Cross‐pollination fruit sets for the 2 years of the study were stable but relatively low. Because the cross‐pollination fruit sets of *B. funingense* were more than 70%, while *B. ambrosia* reached 90% (Chen & Gao, 2011). The natural fruit sets for the 2 years were also low, and there were significant differences between different years. The reason is that part of the inflorescence were eaten by herbivores after pollination, as we also observed all kinds of herbivores eating the flowers in the study populations. In addition, self‐pollination and geitonogamy may be the two main reasons for the low fruit set rate of self‐incompatible orchids (Tremblay et al., 2005). The pollinia removal and deposition are also factors leading to pollination limitation (Tremblay et al., 2005). In *B. funingense*, the pollinia deposition rate being significantly lower than the pollinia removal, which indicates that it also suffers from pollinator‐limitation.

### 4.3 Pollinators and pollination mechanisms

In *Bulbophyllum*, most species have been reported to be pollinated by flies (Christensen, 1994; Dressler, 1993; van der Pijl & Dodson, 1966). *B. funingense* attracts *C. vicina* and *S. carnaria* to lick secretions, and multiple visitors were observed in 2023, but *C. vicina* was its only pollinator. No pollinators were observed in 2022, this may be because the observation time was later in the year, and the flowers at the end of the flowering period were less attractive to pollinators by then.

Plants that are pollinated by animals and in which the animals stay on the flowers and inflorescences for a prolonged time usually present some type of floral mechanism that prevents self‐pollination and geitonogamy (Johnson & Nilsson, 1999; Kropf & Renner, 2008). For example, in *Epidendrum tridactylum*, fly pollinators were caught in a trap while visiting flowers (Pansarin & Pansarin, 2014). The same mechanism also exists in *B. funingense*, although reported in *Govenia* and *Bulbophyllum* (Borba & Semir, 1998; Pansarin, 2008), they did not make detailed statistic.

In our study, *C. vicina* went deeper and deeper to lick the secretions on *B. funingense*'s labellum and eventually got stuck in the access. Detailed statistic were obtained in this study, *C. vicina* trapped for many hours, and even until death. As a result, fly was frightened by being stuck for a long time that inhibits any possibility of immediate visit to another flower and at least presumably reduces the possibility of self‐pollination and geitonogamy (Borba & Semir, 1998; Melo et al., 2010; Pansarin, 2008; Pansarin & Pansarin, 2014). So, *C. vicina* will almost always fly away from the inflorescence after escaping. In just 79 cases counted in this study, *C. vicina* 74 times (94%) immediately flew away from the inflorescence and 64 times (81%) took the pollinia with them. And most of these cases observed where the pollinia was not removed were because the pollinia had been removed before the *C. vicina* visited.

To sum up, *B. funingense* is also self‐incompatible, this floral mechanisms can not only improve the efficiency of pollinia transfer, reduce pollinia waste, and promote pollination success but also reduce the incidence of self‐pollination and avoid sexual conflict to a certain extent.

## 5 CONCLUSION

Many self‐incompatible plants evolve floral mechanisms to reduce sexual conflict (Lloyd & Webb, 1986; Webb & Lloyd, 1986). In fly‐pollinated orchids, the floral mechanism that keeps flies stuck for a long time may avoid self‐pollination (Pansarin, 2008; Pansarin & Pansarin, 2014). *B. funingense* also has this floral mechanism that may avoid sexual conflict to a certain extent. The secretions attract flies to visit the flowers and the flies are then stuck in the access for a long time; this mechanism improves pollinia transfer efficiency, reduces pollinia waste, promotes pollination success, and reduces the incidence of self‐pollination.

## AUTHOR CONTRIBUTIONS

**Sheng Zhang:** Data curation (equal); formal analysis (equal); investigation (equal); writing – original draft (equal); writing – review and editing (equal). **Shi‐Mao Wu:** Investigation (equal); methodology (equal); writing – review and editing (equal). **Jiang‐Yun Gao:** Funding acquisition (equal); writing – original draft (equal); writing – review and editing (equal).

## Supporting information


Video S1

Table S1

## Data Availability

The original contributions presented in the study are included in the article/Tables S1–S6. Data are freely available and deposited in Zenodo (https://doi.org/10.5281/zenodo.10118830).
