# Supplementary material for: Floral mechanisms promote pollination success and reduce the incidence of self‐pollination in a fly‐pollinated self‐incompatible orchid
Source: Ecol Evol. 2024 Apr 24;14(4):e11295. doi: 10.1002/ece3.11295 (PMC11040234; doi:10.1002/ece3.11295)
Supplement: Supplementary file 2 — Table S1 [file ECE3-14-e11295-s002.docx]

**Supporting Information:**

**Floral mechanisms promote pollination success and reduce the incidence of self-pollination in a fly-pollinated self-incompatible orchid**

Sheng Zhang, Shi-Mao Wu and Jiang-Yun Gao*

Institute of Biodiversity, School of Ecology and Environmental Science, Yunnan University, Kunming, Yunnan 650500, China

*Corresponding author

E-mail address: jiangyun.gao@ynu.edu.cn (J.Y. Gao)

Tel. 0086 15368101016

**Supporting Information S1:** Supplemental tables that data statistics results.

**Table of Contents:**

**Table S1:** The floral longevity after different treatments. Page 3.

**Table S2:** The hand-pollinated fruit sets. Page 4.

**Table S3:** The natural fruit sets. Page 6.

**Table S4:** The pollinia removal and deposition rate. Page 10.

**Table S5:** The floral traits and the pollinator size. Page 11.

**Table S6:** The time that pollinators were stuck in the access and pollinator's behavior after escaping from access. Page 13.

**Table S1:** The floral longevity statistics for different treatments (N=20).

| **Inflorescence** | **Unpollinated（d）** | **Self-pollinated（d）** | **Cross-pollinated（d）** |
| --- | --- | --- | --- |
| 1 | 7 | 3 | 4 |
| 2 | 9 | 3 | 3 |
| 3 | 13 | 4 | 3 |
| 4 | 10 | 3 | 3 |
| 5 | 10 | 3 | 4 |
| 6 | 15 | 4 | 3 |
| 7 | 11 | 4 | 4 |
| 8 | 10 | 3 | 3 |
| 9 | 9 | 3 | 3 |
| 10 | 11 | 3 | 3 |
| 11 | 10 | 3 | 4 |
| 12 | 12 | 3 | 3 |
| 13 | 9 | 4 | 4 |
| 14 | 10 | 4 | 3 |
| 15 | 13 | 4 | 3 |
| 16 | 14 | 3 | 3 |
| 17 | 10 | 3 | 4 |
| 18 | 12 | 4 | 3 |
| 19 | 11 | 3 | 3 |
| 20 | 10 | 3 | 3 |

**Table S2:** The hand-pollinated fruit sets.

| **Hand pollination treatments in 2022** | | | | | | | | |
| --- | --- | --- | --- | --- | --- | --- | --- | --- |
| **Bagging（N=27）** | | **Self（N=27）** | | | | **Cross（N=27）** | | |
| **Flowers/fruits** | **Fruit set** | **Flowers/fruits** | | **Fruit set** | | **Flowers/fruits** | | **Fruit set** |
| 2-0 | 0.0000 | 3-0 | | 0.0000 | | 2-2 | | 1.0000 |
| 2-0 | 0.0000 | 2-0 | | 0.0000 | | 2-2 | | 1.0000 |
| 2-0 | 0.0000 | 2-0 | | 0.0000 | | 2-2 | | 1.0000 |
| 3-0 | 0.0000 | 1-0 | | 0.0000 | | 2-1 | | 0.5000 |
| 2-0 | 0.0000 | 1-0 | | 0.0000 | | 2-2 | | 1.0000 |
| 2-0 | 0.0000 | 2-0 | | 0.0000 | | 1-1 | | 1.0000 |
| 2-0 | 0.0000 | 1-0 | | 0.0000 | | 2-2 | | 1.0000 |
| 2-0 | 0.0000 | 2-0 | | 0.0000 | | 2-1 | | 0.5000 |
| 2-0 | 0.0000 | 2-0 | | 0.0000 | | 2-1 | | 0.5000 |
| 3-0 | 0.0000 | 3-0 | | 0.0000 | | 2-2 | | 1.0000 |
| 2-0 | 0.0000 | 2-0 | | 0.0000 | | 2-0 | | 0.0000 |
| 1-0 | 0.0000 | 2-0 | | 0.0000 | | 2-2 | | 1.0000 |
| 2-0 | 0.0000 | 2-0 | | 0.0000 | | 2-2 | | 1.0000 |
|  |  | 2-0 | | 0.0000 | | 2-0 | | 0.0000 |
| **Hand pollination treatments in 2023** | | | | | | | | |
| **Bagging（N=50）** | | **Self（N=50）** | | | **Cross（N=50）** | | | |
| **Flowers/fruits** | **Fruit set** | **Flowers/fruits** | **Fruit set** | | **Flowers/fruits** | | **Fruit set** | |
| 2-0 | 0.0000 | 2-0 | 0.0000 | | 2-1 | | 0.5000 | |
| 2-0 | 0.0000 | 1-0 | 0.0000 | | 2-2 | | 1.0000 | |
| 3-0 | 0.0000 | 2-0 | 0.0000 | | 2-2 | | 1.0000 | |
| 2-0 | 0.0000 | 2-0 | 0.0000 | | 1-0 | | 0.0000 | |
| 1-0 | 0.0000 | 2-0 | 0.0000 | | 2-2 | | 1.0000 | |
| 1-0 | 0.0000 | 3-0 | 0.0000 | | 3-2 | | 0.6667 | |
| 2-0 | 0.0000 | 1-0 | 0.0000 | | 2-1 | | 0.5000 | |
| 2-0 | 0.0000 | 2-0 | 0.0000 | | 2-2 | | 1.0000 | |
| 2-0 | 0.0000 | 2-0 | 0.0000 | | 2-1 | | 0.5000 | |
| 2-0 | 0.0000 | 2-0 | 0.0000 | | 2-2 | | 1.0000 | |
| 3-0 | 0.0000 | 2-0 | 0.0000 | | 3-1 | | 0.3333 | |
| 1-0 | 0.0000 | 2-0 | 0.0000 | | 2-2 | | 1.0000 | |
| 2-0 | 0.0000 | 2-0 | 0.0000 | | 2-1 | | 0.5000 | |
| 2-0 | 0.0000 | 2-0 | 0.0000 | | 2-1 | | 0.5000 | |
| 2-0 | 0.0000 | 2-0 | 0.0000 | | 2-2 | | 1.0000 | |
| 2-0 | 0.0000 | 1-0 | 0.0000 | | 1-1 | | 1.0000 | |
| 3-0 | 0.0000 | 2-0 | 0.0000 | | 2-2 | | 1.0000 | |
| 2-0 | 0.0000 | 2-0 | 0.0000 | | 2-1 | | 0.5000 | |
| 2-0 | 0.0000 | 2-0 | 0.0000 | | 2-2 | | 1.0000 | |
| 2-0 | 0.0000 | 2-0 | 0.0000 | | 2-2 | | 1.0000 | |
| 2-0 | 0.0000 | 2-0 | 0.0000 | | 2-2 | | 1.0000 | |
| 2-0 | 0.0000 | 2-0 | 0.0000 | | 2-0 | | 0.0000 | |
| 2-0 | 0.0000 | 1-0 | 0.0000 | | 1-1 | | 1.0000 | |
| 2-0 | 0.0000 | 2-0 | 0.0000 | | 2-1 | | 0.5000 | |
| 2-0 | 0.0000 | 2-0 | 0.0000 | | 1-0 | | 0.0000 | |
|  |  | 2-0 | 0.0000 | | 2-2 | | 1.0000 | |
|  |  | 1-0 | 0.0000 | |  | |  | |

**Table S3:** The natural fruit sets.

| **The natural fruit set rates in 2022** | | | |
| --- | --- | --- | --- |
| **The number of inflorescence** | **The number of flowers** | **The number of fruits** | **The fruit set rates** |
| 8 | 16 | 0 | 0.0000 |
| 2 | 4 | 0 | 0.0000 |
| 2 | 4 | 0 | 0.0000 |
| 3 | 6 | 0 | 0.0000 |
| 13 | 28 | 0 | 0.0000 |
| 3 | 6 | 0 | 0.0000 |
| 4 | 8 | 0 | 0.0000 |
| 1 | 2 | 0 | 0.0000 |
| 6 | 12 | 1 | 0.0833 |
| 12 | 26 | 1 | 0.0385 |
| 2 | 4 | 0 | 0.0000 |
| 6 | 12 | 0 | 0.0000 |
| 15 | 30 | 0 | 0.0000 |
| 10 | 20 | 1 | 0.0500 |
| 6 | 12 | 0 | 0.0000 |
| 15 | 30 | 1 | 0.0333 |
| 5 | 10 | 0 | 0.0000 |
| 5 | 10 | 0 | 0.0000 |
| 11 | 22 | 0 | 0.0000 |
| 14 | 28 | 0 | 0.0000 |
| 1 | 2 | 0 | 0.0000 |
| 7 | 14 | 0 | 0.0000 |
| 6 | 12 | 0 | 0.0000 |
| 2 | 4 | 0 | 0.0000 |
| 2 | 4 | 1 | 0.2500 |
| 40 | 82 | 1 | 0.0122 |
| 2 | 4 | 0 | 0.0000 |
| 18 | 36 | 0 | 0.0000 |
| 5 | 10 | 0 | 0.0000 |
| 14 | 26 | 0 | 0.0000 |
| 1 | 2 | 0 | 0.0000 |
| 7 | 14 | 0 | 0.0000 |
| 8 | 16 | 0 | 0.0000 |
| 4 | 8 | 0 | 0.0000 |
| 2 | 4 | 0 | 0.0000 |
| 16 | 34 | 5 | 0.1471 |
| 1 | 2 | 0 | 0.0000 |
| 4 | 10 | 0 | 0.0000 |
| 1 | 2 | 0 | 0.0000 |
| 5 | 10 | 0 | 0.0000 |
| 5 | 10 | 0 | 0.0000 |
| 1 | 2 | 0 | 0.0000 |
| 11 | 18 | 0 | 0.0000 |
| 1 | 2 | 0 | 0.0000 |
| 33 | 70 | 10 | 0.1429 |
| 12 | 24 | 0 | 0.0000 |
| 8 | 16 | 1 | 0.0625 |
| 12 | 24 | 1 | 0.0417 |
| 4 | 8 | 0 | 0.0000 |
| 5 | 10 | 0 | 0.0000 |
| 11 | 22 | 0 | 0.0000 |
| 19 | 38 | 0 | 0.0000 |
| 15 | 30 | 0 | 0.0000 |
| 3 | 6 | 0 | 0.0000 |
| 19 | 40 | 0 | 0.0000 |
| 5 | 10 | 0 | 0.0000 |
| 7 | 14 | 0 | 0.0000 |
| 12 | 24 | 0 | 0.0000 |
| 20 | 40 | 0 | 0.0000 |
| 15 | 30 | 0 | 0.0000 |
| 22 | 46 | 0 | 0.0000 |
| 8 | 16 | 0 | 0.0000 |
| 35 | 72 | 1 | 0.0139 |
| 5 | 10 | 1 | 0.1000 |
| 5 | 10 | 0 | 0.0000 |
| 8 | 16 | 0 | 0.0000 |
| 17 | 34 | 2 | 0.0588 |
| 9 | 18 | 2 | 0.1111 |
| 21 | 44 | 1 | 0.0227 |
| 5 | 10 | 0 | 0.0000 |
| 6 | 12 | 1 | 0.0833 |
| 17 | 34 | 0 | 0.0000 |
| 9 | 18 | 0 | 0.0000 |
| 35 | 70 | 1 | 0.0143 |
| 11 | 22 | 0 | 0.0000 |
| 17 | 32 | 0 | 0.0000 |
| 3 | 6 | 0 | 0.0000 |
| 8 | 16 | 0 | 0.0000 |
| 7 | 14 | 0 | 0.0000 |
| 10 | 20 | 0 | 0.0000 |
| 35 | 70 | 1 | 0.0143 |
| 21 | 42 | 0 | 0.0000 |
| 11 | 22 | 0 | 0.0000 |
| 2 | 4 | 0 | 0.0000 |
| 3 | 6 | 1 | 0.1667 |
| 7 | 14 | 0 | 0.0000 |
| 14 | 28 | 1 | 0.0357 |
| **The natural fruit set rates in 2023** | | | |
| **The number of inflorescence** | **The number of flowers** | **The number of fruits** | **The fruit set rates** |
| 6 | 12 | 2 | 0.1667 |
| 10 | 20 | 1 | 0.0500 |
| 49 | 100 | 7 | 0.0700 |
| 20 | 40 | 3 | 0.0750 |
| 17 | 34 | 3 | 0.0882 |
| 60 | 116 | 2 | 0.0172 |
| 11 | 22 | 1 | 0.0455 |
| 15 | 30 | 1 | 0.0333 |
| 12 | 24 | 1 | 0.0417 |
| 51 | 104 | 8 | 0.0769 |
| 9 | 18 | 2 | 0.1111 |
| 27 | 54 | 3 | 0.0556 |
| 31 | 62 | 4 | 0.0645 |
| 21 | 44 | 0 | 0.0000 |
| 13 | 26 | 4 | 0.1538 |
| 24 | 50 | 3 | 0.0600 |
| 41 | 82 | 6 | 0.0732 |
| 49 | 96 | 5 | 0.0521 |
| 22 | 44 | 3 | 0.0682 |
| 10 | 20 | 5 | 0.2500 |
| 13 | 28 | 1 | 0.0357 |
| 25 | 52 | 4 | 0.0769 |
| 11 | 22 | 1 | 0.0455 |
| 16 | 32 | 4 | 0.1250 |
| 26 | 56 | 1 | 0.0179 |
| 23 | 50 | 3 | 0.0600 |
| 53 | 110 | 3 | 0.0273 |
| 31 | 64 | 0 | 0.0000 |
| 48 | 96 | 3 | 0.0313 |
| 17 | 36 | 2 | 0.0556 |
| 32 | 64 | 2 | 0.0313 |
| 7 | 14 | 1 | 0.0714 |
| 52 | 106 | 2 | 0.0189 |
| 10 | 20 | 3 | 0.1500 |
| 46 | 90 | 15 | 0.1667 |
| 14 | 30 | 1 | 0.0333 |
| 30 | 60 | 0 | 0.0000 |
| 21 | 40 | 1 | 0.0250 |
| 38 | 80 | 5 | 0.0625 |

**Table S4:** The pollinia removal and deposition rate.

| **Flowers** | **Pollinia removed** | **Removal rate** | **Pollinia deposited** | **Deposition rate** |
| --- | --- | --- | --- | --- |
| 70 | 23 | 0.3286 | 3 | 0.0429 |
| 55 | 17 | 0.3091 | 1 | 0.0182 |
| 27 | 9 | 0.3333 | 1 | 0.0370 |
| 20 | 8 | 0.4000 | 3 | 0.1500 |
| 22 | 4 | 0.1818 | 0 | 0.0000 |
| 15 | 6 | 0.4000 | 2 | 0.1333 |
| 18 | 2 | 0.1111 | 2 | 0.1111 |
| 14 | 1 | 0.0714 | 0 | 0.0000 |
| 14 | 1 | 0.0714 | 0 | 0.0000 |
| 25 | 10 | 0.4000 | 3 | 0.1200 |
| 23 | 9 | 0.3913 | 0 | 0.0000 |
| 13 | 3 | 0.2308 | 0 | 0.0000 |
| 13 | 2 | 0.1538 | 0 | 0.0000 |
| 26 | 7 | 0.2692 | 1 | 0.0385 |
| 33 | 11 | 0.3333 | 3 | 0.0909 |
| 25 | 6 | 0.2400 | 0 | 0.0000 |
| 22 | 4 | 0.1818 | 2 | 0.0909 |
| 45 | 15 | 0.3333 | 5 | 0.1111 |
| 20 | 12 | 0.6000 | 2 | 0.1000 |
| 15 | 9 | 0.6000 | 1 | 0.0667 |
| 19 | 5 | 0.2632 | 1 | 0.0526 |
| 10 | 3 | 0.3000 | 1 | 0.1000 |
| 11 | 5 | 0.4545 | 0 | 0.0000 |
| 17 | 6 | 0.3529 | 0 | 0.0000 |
| 11 | 6 | 0.5455 | 0 | 0.0000 |
| 24 | 6 | 0.2500 | 0 | 0.0000 |
| 19 | 7 | 0.3684 | 0 | 0.0000 |
| 17 | 11 | 0.6471 | 0 | 0.0000 |
| 26 | 8 | 0.3077 | 1 | 0.0385 |
| 22 | 9 | 0.4091 | 1 | 0.0455 |
| 38 | 19 | 0.5000 | 1 | 0.0263 |
| 19 | 3 | 0.1579 | 1 | 0.0526 |
| 23 | 8 | 0.3478 | 1 | 0.0435 |
| 29 | 10 | 0.3448 | 2 | 0.0690 |
| 38 | 15 | 0.3947 | 2 | 0.0526 |
| 40 | 8 | 0.2000 | 1 | 0.0250 |
| 46 | 10 | 0.2174 | 0 | 0.0000 |
| 11 | 3 | 0.2727 | 0 | 0.0000 |
| 22 | 6 | 0.2727 | 0 | 0.0000 |
| 27 | 7 | 0.2593 | 0 | 0.0000 |

**Table S5:** The floral traits and the pollinator size.

|  |  | ***Calliphora vicina*** | | |
| --- | --- | --- | --- | --- |
| **Number** | **Repeated measure** | **Thorax height(mm)** | **Thorax width(mm)** | **Body length(mm)** |
| **1** | 1 | 4.25 | 4.52 | 10.21 |
|  | 2 | 4.23 | 4.55 | 10.19 |
|  | 3 | 4.27 | 4.56 | 10.17 |
| **2** | 1 | 4.34 | 4.86 | 10.05 |
|  | 2 | 4.34 | 4.83 | 10.05 |
|  | 3 | 4.32 | 4.85 | 10.07 |
| **3** | 1 | 4.12 | 4.25 | 9.81 |
|  | 2 | 4.19 | 4.30 | 9.97 |
|  | 3 | 4.16 | 4.26 | 9.95 |
| **4** | 1 | 4.36 | 4.64 | 9.97 |
|  | 2 | 4.40 | 4.67 | 9.98 |
|  | 3 | 4.34 | 4.66 | 9.99 |
| **5** | 1 | 4.29 | 4.51 | 9.71 |
|  | 2 | 4.27 | 4.55 | 9.75 |
|  | 3 | 4.25 | 4.56 | 9.70 |
| **6** | 1 | 4.52 | 5.10 | 10.15 |
|  | 2 | 4.53 | 5.17 | 10.14 |
|  | 3 | 4.55 | 5.20 | 10.12 |
|  |  | ***Sarcophaga carnaria*** | | |
| **Number** | **Repeated measure** | **Thorax height(mm)** | **Thorax width(mm)** | **Body length(mm)** |
| **1** | 1 | 5.07 | 4.46 | 12.65 |
|  | 2 | 5.02 | 4.50 | 12.67 |
|  | 3 | 5.03 | 4.49 | 12.64 |
| **2** | 1 | 4.61 | 4.74 | 12.21 |
|  | 2 | 4.64 | 4.78 | 12.19 |
|  | 3 | 4.60 | 4.79 | 12.17 |
| **3** | 1 | 4.70 | 4.45 | 12.03 |
|  | 2 | 4.72 | 4.50 | 12.05 |
|  | 3 | 4.74 | 4.51 | 12.07 |
| **4** | 1 | 4.62 | 4.39 | 11.81 |
|  | 2 | 4.62 | 4.41 | 11.97 |
|  | 3 | 4.65 | 4.40 | 11.95 |
| **5** | 1 | 4.41 | 4.38 | 11.97 |
|  | 2 | 4.46 | 4.41 | 11.98 |
|  | 3 | 4.40 | 4.40 | 11.99 |
| **6** | 1 | 4.15 | 3.73 | 10.71 |
|  | 2 | 4.19 | 3.70 | 10.75 |
|  | 3 | 4.14 | 3.68 | 10.70 |
| **7** | 1 | 4.59 | 4.74 | 12.15 |
|  | 2 | 4.63 | 4.76 | 12.14 |
|  | 3 | 4.56 | 4.71 | 12.12 |
|  |  | **Floral traits** | | |
| **Number** | **Repeated measure** | **Access height(mm)** | **Access width(mm)** | **Access length(mm)** |
| **1** | 1 | 4.30 | 8.49 | 8.62 |
|  | 2 | 4.28 | 8.17 | 8.47 |
|  | 3 | 4.31 | 8.02 | 8.51 |
| **2** | 1 | 4.32 | 8.13 | 8.64 |
|  | 2 | 4.31 | 8.06 | 8.68 |
|  | 3 | 4.30 | 8.12 | 8.78 |
| **3** | 1 | 4.24 | 8.42 | 8.80 |
|  | 2 | 4.23 | 8.31 | 8.65 |
|  | 3 | 4.28 | 8.42 | 8.56 |
| **4** | 1 | 4.36 | 8.19 | 8.89 |
|  | 2 | 4.34 | 8.20 | 8.92 |
|  | 3 | 4.36 | 8.16 | 8.71 |
| **5** | 1 | 4.32 | 8.14 | 8.86 |
|  | 2 | 4.35 | 8.12 | 8.84 |
|  | 3 | 4.36 | 8.06 | 8.70 |
| **6** | 1 | 4.36 | 8.01 | 8.75 |
|  | 2 | 4.34 | 8.12 | 8.50 |
|  | 3 | 4.37 | 8.09 | 8.73 |
| **Note: To eliminate errors, each indicator of fly and floral traits was measured three times** | | | | |
|  |  |  |  |  |
|  |  |  |  |  |

**Table S6:** The time that pollinators were stuck in the access and pollinator's behavior after escaping from access.

|  | The records of Calliphora vicina getting stuck in access  (" Yes =1 ", "No =2") | | |
| --- | --- | --- | --- |
| **Records** | **Stuck time** | **Go away from inflorescence after escaping** | **Carry pollinia** |
| 1 | ＞2h | 1 | 1 |
| 2 | ＞2h | 1 | 1 |
| 3 | 1h-2h | 1 | 2 |
| 4 | ＞2h | 1 | 1 |
| 5 | ＞2h | 1 | 1 |
| 6 | ＞2h | 1 | 1 |
| 7 | ＞2h | 1 | 1 |
| 8 | 5min-1h | 1 | 1 |
| 9 | 5min-1h | 1 | 2 |
| 10 | ＞2h | 1 | 1 |
| 11 | ＞2h | 1 | 1 |
| 12 | ＞2h | 1 | 1 |
| 13 | ＞2h | 1 | 1 |
| 14 | 1h-2h | 1 | 1 |
| 15 | 5min-1h | 1 | 2 |
| 16 | 1h-2h | 1 | 1 |
| 17 | 5min-1h | 1 | 1 |
| 18 | 5min-1h | 1 | 1 |
| 19 | ＞2h | 1 | 1 |
| 20 | ＞2h | 1 | 1 |
| 21 | ＞2h | 1 | 1 |
| 22 | ＞2h | 1 | 1 |
| 23 | 5min-1h | 1 | 2 |
| 24 | ＞2h | 1 | 1 |
| 25 | ＞2h | 1 | 1 |
| 26 | ＞2h | 1 | 1 |
| 27 | ＞2h | 1 | 1 |
| 28 | 5min-1h | 1 | 2 |
| 29 | ＜5min | 2 | 1 |
| 30 | ＞2h | 1 | 1 |
| 31 | 1h-2h | 1 | 1 |
| 32 | 1h-2h | 1 | 1 |
| 33 | 5min-1h | 1 | 2 |
| 34 | ＞2h | 1 | 1 |
| 35 | 1h-2h | 1 | 1 |
| 36 | ＞2h | 1 | 1 |
| 37 | ＞2h | 1 | 1 |
| 38 | 1h-2h | 1 | 1 |
| 39 | ＞2h | 1 | 1 |
| 40 | ＞2h | 1 | 1 |
| 41 | ＞2h | 1 | 1 |
| 42 | ＞2h | 1 | 1 |
| 43 | ＞2h | 1 | 1 |
| 44 | 5min-1h | 1 | 1 |
| 45 | 5min-1h | 1 | 2 |
| 46 | 1h-2h | 1 | 1 |
| 47 | 1h-2h | 1 | 1 |
| 48 | 5min-1h | 1 | 1 |
| 49 | ＞2h | 1 | 1 |
| 50 | 5min-1h | 1 | 1 |
| 51 | ＞2h | 1 | 1 |
| 52 | ＜5min | 2 | 2 |
| 53 | ＞2h | 1 | 1 |
| 54 | ＞2h | 1 | 1 |
| 55 | 1h-2h | 1 | 1 |
| 56 | 5min-1h | 1 | 2 |
| 57 | 1h-2h | 1 | 1 |
| 58 | 5min-1h | 1 | 2 |
| 59 | ＞2h | 1 | 1 |
| 60 | 1h-2h | 1 | 1 |
| 61 | ＜5min | 2 | 2 |
| 62 | ＞2h | 1 | 1 |
| 63 | 1h-2h | 1 | 1 |
| 64 | ＞2h | 1 | 1 |
| 65 | ＞2h | 1 | 2 |
| 66 | ＞2h | 1 | 1 |
| 67 | ＞2h | 1 | 1 |
| 68 | ＞2h | 1 | 1 |
| 69 | ＞2h | 1 | 1 |
| 70 | 5min-1h | 1 | 2 |
| 71 | 1h-2h | 1 | 1 |
| 72 | ＜5min | 2 | 2 |
| 73 | ＞2h | 1 | 1 |
| 74 | 1h-2h | 1 | 1 |
| 75 | ＜5min | 2 | 1 |
| 76 | ＞2h | 1 | 1 |
| 77 | 1h-2h | 1 | 2 |
| 78 | 1h-2h | 1 | 1 |
| 79 | ＞2h | 1 | 1 |
